# Iatrogenic Gastric Perforation Following Emergency Endoscopic Decompression for Obstructive Shock Caused by an Incarcerated Hiatal Hernia: A Case Report

**DOI:** 10.7759/cureus.107263

**Published:** 2026-04-17

**Authors:** Yo Sugimori, Fujii Mahiro, Kikukawa Tetsuei, Hajime Sunagozaka, Sawada Kouichiro, Yoshida Masahiro

**Affiliations:** 1 Department of Emergency and Critical Care Medicine, JA Toyama Kouseiren Takaoka Hospital, Takaoka, JPN; 2 Department of Gastroenterology, JA Toyama Kouseiren Takaoka Hospital, Takaoka, JPN; 3 Department of Surgery, JA Toyama Kouseiren Takaoka Hospital, Takaoka, JPN

**Keywords:** gastric volvulus, hiatal hernia, iatrogenic gastric perforation, obstructive shock, tension gastrothorax, upper endoscopy

## Abstract

Hiatal hernias are a common condition, particularly in the elderly. While often asymptomatic, they can lead to life-threatening complications such as incarceration, strangulation, and gastric volvulus. Obstructive shock due to cardiac compression is a rare but critical presentation.

We present the case of a 77-year-old Japanese man with a history of renal transplantation who was admitted for conservative management of an incarcerated hiatal hernia. On the second day of admission, he developed profound shock, requiring ICU admission. A malpositioned nasogastric tube had failed to decompress the stomach, which was twisted due to volvulus. Point-of-care ultrasound and computed tomography confirmed a massive paraesophageal hernia causing severe cardiac compression, leading to a diagnosis of obstructive shock. An emergency endoscopic decompression was performed due to refractory shock and the inability to reposition the gastric tube. While initial gastric suction resulted in immediate hemodynamic improvement, the procedure was complicated by iatrogenic gastric perforation, leading to recurrent shock. After stabilization with conservative management, definitive laparoscopic hernia repair was performed on day 11 and he was eventually discharged home.

Incarcerated hiatal hernia can cause life-threatening obstructive shock. While immediate gastric decompression is crucial, endoscopic intervention carries a significant risk of perforation due to the ischemic and fragile gastric wall. If endoscopy is deemed unavoidable for life-saving decompression, it should be limited to minimal suction to stabilize the patient for definitive surgical repair.

## Introduction

A hiatal hernia is defined as the herniation of a portion of the stomach from the abdominal cavity into the mediastinum through a widened esophageal hiatus in the diaphragm. Its prevalence increases significantly with age, making it a common finding in the elderly population [1]. The vast majority (over 90%) are Type I, or "sliding" hernias. Type II-IV paraesophageal hernias (PEH) are less common but carry a significant risk of mechanical complications due to the gastroesophageal junction remaining in its normal position while the gastric fundus herniates into the chest [2].

Incarceration of a PEH can initiate a life-threatening cascade of events, including ischemia from strangulation, gastric volvulus, tissue necrosis, and ultimately, perforation [3]. While these complications are well-documented, a rarer presentation is obstructive shock resulting from direct cardiac compression by the massively dilated, herniated stomach [4]. Standard management for an incarcerated hernia is emergency surgical repair [5]. This case report describes a rare presentation of obstructive shock caused by an incarcerated hiatal hernia and highlights the critical complication of iatrogenic gastric perforation during a necessary, life-saving endoscopic decompression. The purpose of this report is to raise awareness of this clinical scenario and discuss the management challenges it presents.

## Case presentation

A 77-year-old Japanese man with a history of chronic kidney disease status post living-donor renal transplantation, myocardial infarction status post-percutaneous coronary intervention, and rectal cancer status post-surgery presented to our emergency department with a one-day history of abdominal pain and vomiting. His maintenance medications included tacrolimus, mycophenolate mofetil, prednisolone 5 mg/day, and prasugrel.

**Figure 1:**
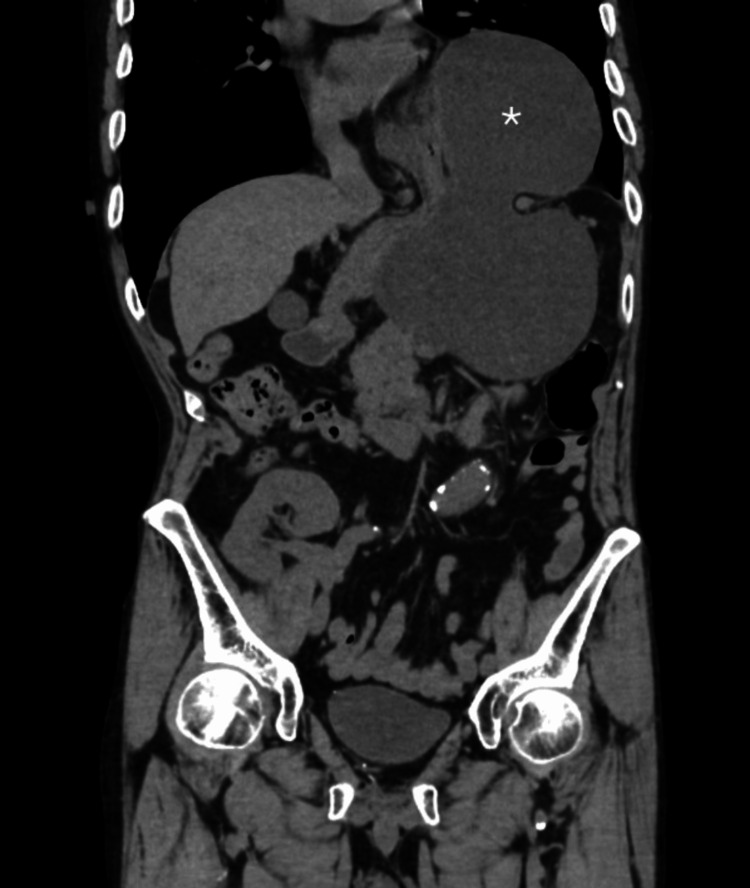
CT demonstrating an incarcerated hiatal hernia Coronal CT on admission demonstrating an incarcerated hiatal hernia with herniation. The asterisk (*) indicates the herniated stomach in the left thoracic cavity.

On initial presentation, his vital signs were stable. A computed tomography (CT) scan revealed an incarcerated paraesophageal hiatal hernia with evidence of gastric volvulus (Figure 1).

A nasogastric (NG) tube was inserted for decompression with a plan for conservative management and inpatient observation.

Despite NG tube placement, he continued to vomit. On the second day of admission, his condition deteriorated rapidly with tachycardia, vomiting, and altered mental status. As his condition was deemed life-threatening, the Rapid Response Team was activated and an urgent clinical evaluation was initiated. On assessment, his heart rate was 180 bpm, blood pressure was unmeasurable, and his Glasgow Coma Scale (GCS) score was five (E3V1M1). He was immediately transferred to the intensive care unit (ICU) for resuscitation.

**Figure 2:**
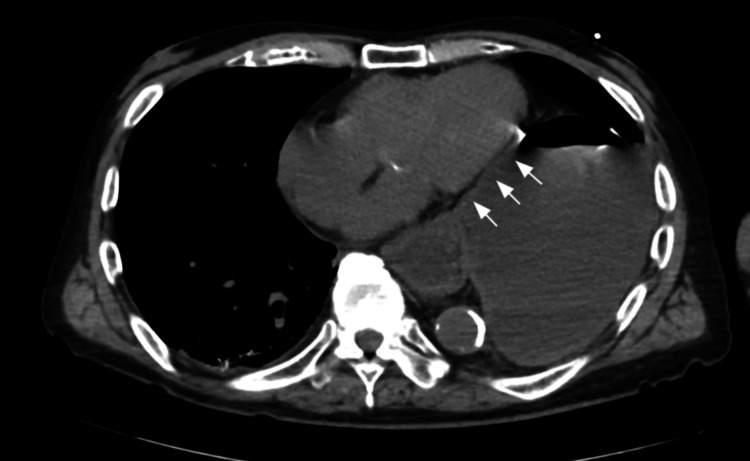
Axial CT at the time of hemodynamic collapse. The distended stomach compresses the heart, as indicated by the arrows.

On ICU admission, physical examination revealed decreased breath sounds on the left, a soft but distended abdomen with epigastric tenderness, and no peripheral edema. Arterial blood gas analysis showed a mixed acid-base disorder with metabolic alkalosis from vomiting and severe lactic acidosis (pH 7.411, partial pressure of carbon dioxide (pCO_2_) 36 mmHg, partial pressure of oxygen (pO_2_) 261 mmHg, bicarbonate (HCO_3_) 22.3 mmol/L, lactate 17 mmol/L). Point-of-care ultrasound (POCUS) demonstrated a collapsed inferior vena cava (IVC) and significant external compression of the heart by the stomach, resulting in collapsed cardiac chambers. A repeat CT scan confirmed a massive incarcerated paraesophageal hernia severely compressing and displacing the heart (Figure 2).

It also revealed that the NG tube was coiled in the distal esophagus, proximal to the twisted gastroesophageal junction, thus failing to decompress the stomach. Laboratory findings were significant for Na at 151 mmol/L, blood urea nitrogen (BUN) at 49.6 mg/dL, creatinine (Cr) at 3.57 mg/dL, creatine kinase (CK) at 15,670 U/L, and white blood cell count (WBC) at 15,800/μL.

**Figure 3:**
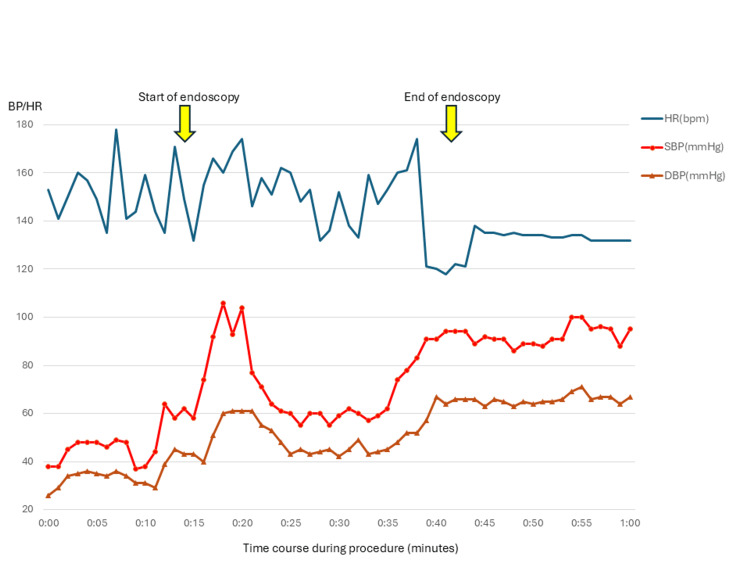
Hemodynamic changes during endoscopic decompression. Blood pressure rapidly increased immediately after gastric decompression, confirming the diagnosis of obstructive shock. Blood pressure decreased again with insufflation during endoscopic manipulation and subsequently stabilized after completion of the procedure.

Following fluid resuscitation, the patient's systolic blood pressure (SBP) transiently improved to 90 mmHg and his GCS improved to 13 (E3V4M6), but he quickly devolved into refractory shock despite aggressive fluid administration and vasopressor support. Given the clear evidence of obstructive shock, immediate gastric decompression was deemed a priority. After endotracheal intubation, emergency upper gastrointestinal endoscopy was performed to promptly resolve the obstructive shock. With norepinephrine infusing at 0.15 mcg/kg/min, his SBP was approximately 50 mmHg. Immediately upon endoscopic suction of the gastric contents, his SBP rapidly improved to 100 mmHg, confirming the diagnosis of obstructive shock. However, during the remaining procedures, including derotation of the volvulus, insufflation again caused his blood pressure to decrease to 60 mmHg (Figure 3).

**Figure 4:**
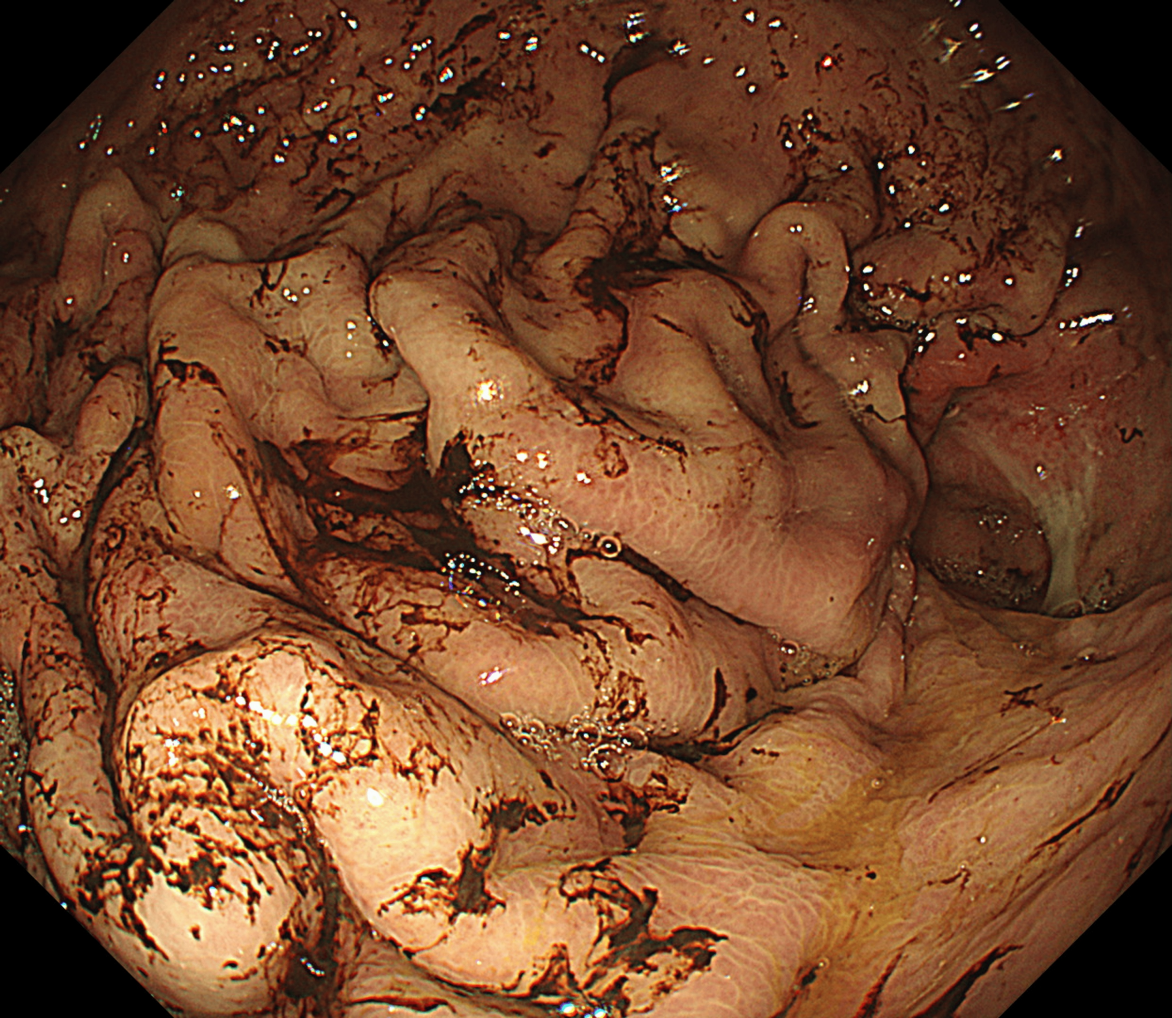
Upper gastrointestinal endoscopic findings. Upper gastrointestinal endoscopy demonstrates globally impaired gastric mucosal perfusion without evidence of overt necrosis.

Endoscopy showed poor mucosal perfusion without necrosis (Figure 4).

**Figure 5:**
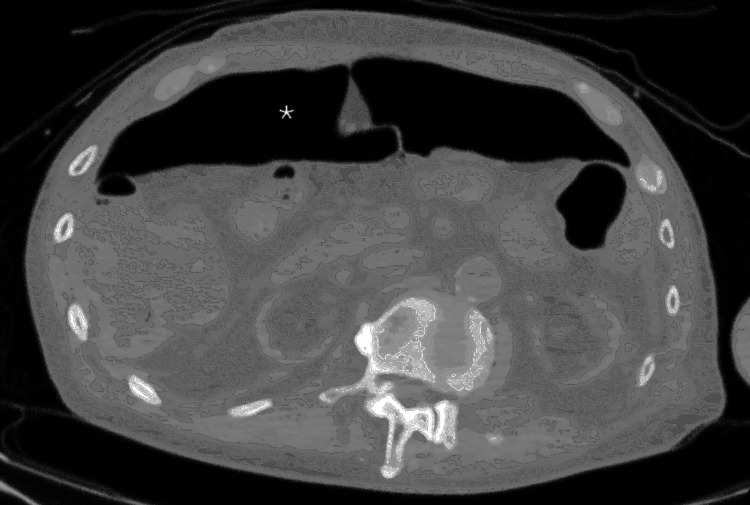
CT after endoscopic intervention. Axial plain CT demonstrates pneumoperitoneum, as indicated by the asterisk, without associated intra-abdominal fluid collection.

At the conclusion of the procedure, the patient’s abdomen was noted to be severely distended. An abdominal X-ray was suspicious for pneumoperitoneum, and a subsequent CT scan confirmed a massive amount of free air without any fluid collection, consistent with gastric perforation (Figure 5).

Although an emergency laparotomy was initially considered, conservative management was elected because the CT scan demonstrated isolated pneumoperitoneum without any associated free fluid or organized collections, and his hemodynamics showed rapid stabilization.

He was managed conservatively and was successfully extubated on ICU day four. On ICU day 11, he underwent a laparoscopic repair of the giant hiatal hernia and micro-perforation. Intraoperatively, dense adhesions and a micro-abscess were noted. The stomach was massively dilated and could only be reduced into the abdominal cavity after intraoperative endoscopic decompression. The hernia sac was fully dissected, the crural defect was closed, and a partial anterior fundoplication with gastropexy was performed. The gastric wall was noted to be extremely fragile, with multiple serosal tears occurring during manipulation.

His postoperative course was uneventful. He was discharged from the ICU on day 13 and discharged home without any complications on hospital day 33. At the six-month follow-up, there was no evidence of recurrence.

## Discussion

Incarceration is a rare but severe complication of hiatal hernias, primarily occurring in type III and IV PEH, and is the cause of death in approximately 75% of fatal cases [6]. The pathophysiology commonly follows a perilous cascade: mechanical incarceration and obstruction can produce gastric distension and, in many cases, gastric volvulus within the confined hernia sac. Severe volvulus or prolonged obstruction may cause vascular compromise, leading to strangulation, ischemia, and ultimately necrosis and perforation if not promptly treated [7]. Shock in this setting can be multifactorial, including septic shock from mediastinitis or peritonitis, and hypovolemic shock from fluid losses, and obstructive shock from cardiac compression as demonstrated by an immediate hemodynamic response to gastric decompression in some case reports [4,8]. In this case, the presentation of life-threatening obstructive shock due to an incarcerated hiatal hernia created a uniquely urgent clinical scenario. To our knowledge, a PubMed search using the keywords 'obstructive shock' and 'hiatal hernia' identified only six cases reported in the English literature as of March 2026.

The iatrogenic perforation observed was likely attributable to the extreme fragility of the ischemic gastric wall, which is well-documented as a risk factor for perforation during endoscopic manipulation in the setting of acute incarceration and compromised perfusion [9,10].

Current evidence and guidelines, including those from the Society of American Gastrointestinal and Endoscopic Surgeons, establish emergency surgery as the definitive treatment for incarcerated hiatal hernia with clinical instability or evidence of ischemia, perforation, or necrosis [5]. However, in rare cases of profound obstructive shock, immediate decompression may be necessary to stabilize hemodynamics prior to surgical intervention. Nasogastric tube placement is the preferred initial method, but when this fails, endoscopic decompression may be considered as a temporizing measure [9,10].

Recent cohort studies demonstrate that endoscopic decompression can allow for semi-elective surgery in a majority of acute presentations, reducing morbidity associated with emergent operations [10,11]. Nevertheless, the risk of perforation is significantly increased in the context of ischemic or necrotic gastric tissue. Therefore, endoscopic intervention should be strictly limited to the minimum necessary suction for hemodynamic stabilization, avoiding attempts at full derotation or aggressive manipulation. This approach is supported by both clinical experience and published outcomes, which indicate that minimal intervention may mitigate the risk of perforation while serving as a critical bridge to definitive surgical repair [9,10]. Since this is a single case report, the findings regarding the utility of endoscopy cannot be generalized.

## Conclusions

This case highlights that incarcerated hiatal hernia can rarely cause life-threatening obstructive shock due to cardiac compression by a massively distended stomach. Prompt gastric decompression is essential for hemodynamic stabilization. Although nasogastric tube placement should be attempted first, endoscopic decompression may be considered when this approach fails. However, because the ischemic gastric wall is extremely fragile, endoscopic manipulation should be minimized to reduce the risk of iatrogenic perforation.
